# Functional Analysis of Conserved Non-Coding Regions Around the Short Stature *hox* Gene (*shox*) in Whole Zebrafish Embryos

**DOI:** 10.1371/journal.pone.0021498

**Published:** 2011-06-24

**Authors:** Emma J. Kenyon, Gayle K. McEwen, Heather Callaway, Greg Elgar

**Affiliations:** 1 Wellcome Trust Sanger Institute, Wellcome Trust Genome Campus, Cambridge, Cambridgeshire, United Kingdom; 2 Vertebrate Genomics, Max Planck Institute for Molecular Genetics, Berlin, Germany; 3 Zebrafish Facility, University College London, London, Greater London, United Kingdom; 4 MRC National Institute for Medical Research, London, Greater London, United Kingdom; Centre for Genomic Regulation, Spain

## Abstract

**Background:**

Mutations in the *SHOX* gene are responsible for Leri-Weill Dyschondrosteosis, a disorder characterised by mesomelic limb shortening. Recent investigations into regulatory elements surrounding *SHOX* have shown that deletions of conserved non-coding elements (CNEs) downstream of the *SHOX* gene produce a phenotype indistinguishable from Leri-Weill Dyschondrosteosis. As this gene is not found in rodents, we used zebrafish as a model to characterise the expression pattern of the *shox* gene across the whole embryo and characterise the enhancer domains of different CNEs associated with this gene.

**Methodology/Principal Findings:**

Expression of the *shox* gene in zebrafish was identified using *in situ* hybridization, with embryos showing expression in the blood, putative heart, hatching gland, brain pharyngeal arch, olfactory epithelium, and fin bud apical ectodermal ridge. By identifying sequences showing 65% identity over at least 40 nucleotides between *Fugu*, human, dog and opossum we uncovered 35 CNEs around the *shox* gene. These CNEs were compared with CNEs previously discovered by Sabherwal *et al.*, *r*esulting in the identification of smaller more deeply conserved sub-sequence. Sabherwal *et al.*'s CNEs were assayed for regulatory function in whole zebrafish embryos resulting in the identification of additional tissues under the regulatory control of these CNEs.

**Conclusion/Significance:**

Our results using whole zebrafish embryos have provided a more comprehensive picture of the expression pattern of the *shox* gene, and a better understanding of its regulation via deeply conserved noncoding elements. In particular, we identify additional tissues under the regulatory control of previously identified *SHOX* CNEs. We also demonstrate the importance of these CNEs in evolution by identifying duplicated *shox* CNEs and more deeply conserved sub-sequences within already identified CNEs.

## Introduction

Mutations in the short stature *HOX* gene, *SHOX* have been shown to be responsible for the dominantly inherited skeletal dysplasia Leri-Weill Dyschondrosteosis (LWD). LWD is characterised by disproportionate short stature with mesomelic limb shortening [1]. This disorder is the result of haploinsufficiency of the *SHOX* gene, which is found in the pseudoautosomal region at the telomere of the short arm of the X and Y chromosomes. *SHOX* was discovered when looking for a Turner syndrome short-stature gene in the Xp-Yp pseudoautosomal region (PAR1). Genes within this region escape X-inactivation in females and participate in obligate recombination during male meiosis. This results in LWD being an apparently “autosomal" dominant disorder. Turner syndrome is also characterised by short stature but is frequently associated with a variable spectrum of somatic features, including ovarian failure, heart and renal abnormalities, micrognathia, cubitus valgus, high-arched palate, short metacarpals and Madelung deformity [2], [3]. SHOX is thus haploinsufficient in females with 45,X Turner syndrome, accounting for approximately two-thirds of the characteristic growth deficit [4], [5].

In human, the *SHOX* gene has two isoforms, with *SHOXA* expressed in skeletal muscle, placenta, pancreas, heart and bone marrow fibroblasts and *SHOXB* transcripts restricted to fetal kidney, skeletal muscle and bone marrow fibroblasts [2]. In the whole embryo, *SHOXA* has been shown to be expressed in the central part of both upper and lower limbs (UL and LL), and in the first pharyngeal arch (1st PA) [6] of a lateral, sagittal section of a CS16 human embryo.

In cases such as these, it would be expected that a mouse model would be used to further characterise the function of the gene but, interestingly, this gene is not found in rodents. Therefore, the only non-human research into this gene has been carried out in chick. *In situ* hybridisation in whole chick embryos has shown expression in branchial arches, hindlimb buds and neural tube of the developing chick embryo [7], with some expression in the mesenchyme overlaying the eye. Closer inspection of the expression in the limb found *SHOX* expressed in the central region of the early limb bud, leaving a rim of non-expressing cells around it. Sectioning showed the expression to be in a thin layer of mesenchyme, just under the ectoderm. Later stages showed expression is restricted to the proximal two thirds of the limb bud and eventually becomes expressed in the digital rays, with stronger expression dorsally, and stripes of expression in the muscle and strong expression under the dermis.

A second member of the *SHOX* family, *SHOX2*, is present in the rodent lineage and also shows expression in the limb in addition to other tissues. In human, *SHOX2* has a subtly different expression pattern to *SHOX*. Where *SHOX* expression in the human limb is observed as a band across the limb and then around the pre-cartilaginous structure of the bones of the elbow joint, with expression becoming confined to the middle portion of the arm, most highly in the perichondrial tissue. The expression of *SHOX2* is seen in the dorsal region of the lower limb. In the forelimbs *SHOX2* expression is more proximal to that of *SHOX* and is also observed in the dorsal root ganglia [8]. In mouse limb *Shox2* expression is observed in mesodermal cells on the dorsal side of the limb bud with expression intensifying in the mesoderm of the progress zone and in undifferentiated mesoderm condensing around the ossification centres. It is also found in ectodermal tissue including brain, spinal cord, and ganglia (in otic) with the highest levels of expression found in mesodermal tissues of the face involved in nose and palate formation, the developing eyelid and tissue surrounding the optic nerve, as well as in the developing heart mesoderm, although heart expression is restricted to the developing outflow track and the developing aorta [9].

Investigations into regulatory elements around *SHOX* have found that deletion of conserved non-coding elements (CNEs) downstream of the *SHOX* gene produces a phenotype indistinguishable from patients with mutations in the *SHOX* coding region and so resulting in Leri-Weill Dyschondrosteosis. These CNEs were electroporated into chick limbs and shown to drive expression in the limb bud [10]. This assay, by its nature, will only show expression in the limb bud. By using zebrafish as a model, we aimed to characterise the expression pattern of the *shox* gene across the whole embryo and also characterise the enhancer domains of different CNEs associated with this gene. Finally, by using elements from both *Fugu* and human we aim to develop a better understanding of the evolution of these CNEs. This will result in a more comprehensive understanding of the range of expression in this gene, and the regulatory elements that are, at least in part, responsible for directing that expression.

## Results

### Identification of CNEs at the *shox* locus

Conserved non-coding elements (CNEs) around the *shox* gene were identified from a multiple alignment of the human, dog, opossum and *Fugu* loci using MLAGAN [11]. These elements were submitted to the CONDOR database [12] and given unique identifiers, for example CRCNE00011095. Table 1 shows the location of these CNEs in the human genome. Sabherwal *et al.* [10] also identified CNEs downstream of the human *SHOX* gene, within a 200 kb minimally deleted region associated with short stature. Sabherwal *et al.* named these CNE4, CNE5, CNE6, CNE7, CNE8 and CNE9, with CNE7, CNE8 and CNE9 being shown to be deleted in patients with short stature. These Sabherwal CNEs are longer than those identified in the CONDOR database, such that each one can encompass an area that includes multiple CONDOR CNEs. This is illustrated in table 1, where, for example, Sabherwal's CNE5 encompasses CONDOR CNEs CRCNE00011075, CRCNE00011096, CRCNE00011097, CRCNE00011098 and CRCNE00011099. In this study we tested Sabherwal *et al*'s human CNEs (hCNE) and the orthologous *Fugu* (fCNE) region in the zebrafish embryo.

**Table 1:** Positions of CNEs found around the Human *shox* gene using Human GRCh37 Ensembl release 61 (Feb 2011).

	Human genomic	
Condor CNE ID	co-ordinates	Sabherwal CNEs
CRCNE00011081	395932–396139	
CRCNE00011074	398484–398605	
CRCNE00011082	398646–398808	
CRCNE00011083	421661–421749	
CRCNE00011084	421789–421848	
**CRCNE00011085**	433506–433625	
CRCNE00011139	443510–443605	
CRCNE00011086	455000–455084	
CRCNE00011088	460789–460848	
CRCNE00011089	516667–516966	
**SHOX Gene**	585079–620146	
CRCNE00011090	591474–591537	
CRCNE00011115	598067–598108	
CRCNE00011091	598664–598921	
**CRCNE00011094**	612250–612333	
CRCNE00011095	714364–714449	CNE4
CRCNE00011075	750835–750884	CNE5
CRCNE00011096	751022–751081	
CRCNE00011097	751185–751471	
**CRCNE00011098**	751521–751685	
CRCNE00011099	751698–751757	
CRCNE00011100	766109–766197	CNE6
CRCNE00011101	780784–780955	CNE7
CRCNE00011102	781023–781132	
CRCNE00011103	811745–812051	CNE8
**CRCNE00011104**	835191–835339	CNE9
CRCNE00011105	835390–835434	
CRCNE00011106	866772–866831	
CRCNE00011107	934004–934039	
CRCNE00011108	934182–934302	
CRCNE00011109	963804–963863	
**CRCNE00011110**	995756–995967	
**CRCNE00011111**	1194952–1194993	
CRCNE00011112	1199641–1199687	
CRCNE00011113	1211415–1211449	
CRCNE00011114	1211533–1211776	

**Figure 1:**
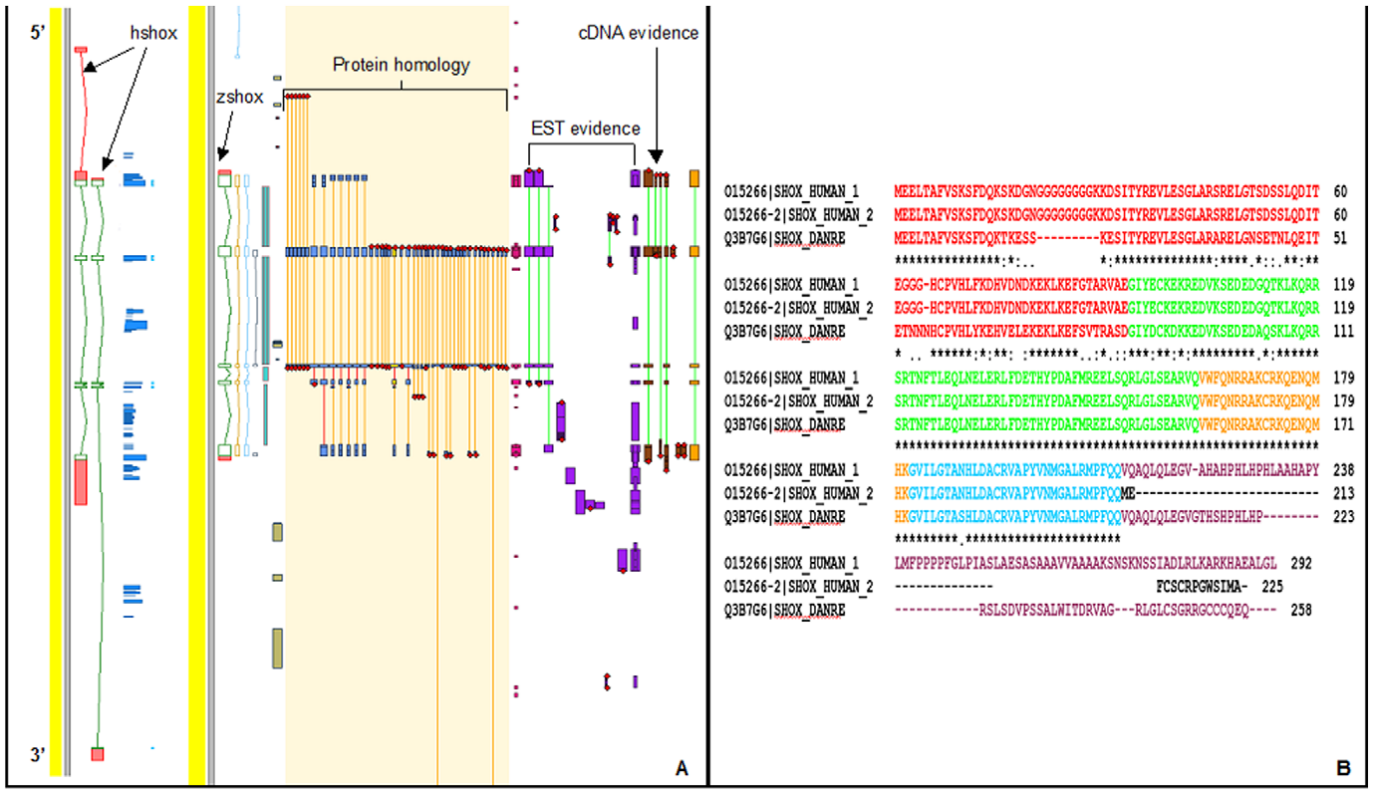
Comparison of zebrafish and human *shox* gene and protein alignments: (A) Screenshot of Otterlace annotation software used by the Wellcome Trust Sanger Institute to manually annotate the human and zebrafish genome for the Vega and Ensembl genome browsers. Protein evidence is shown in blue, EST evidence is shown in purple, and mRNA evidence is shown in brown with the boxes denoting exons. Human and zebrafish *shox* gene coding variants are shown as green and red boxes with green denoting coding areas and red denoting non-coding parts. (B) Protein alignment comparing the zebrafish protein (uniprot ID Q3B7G6) with the human protein isoforms (uniprot ID 015266 and 015266-2). Separate exons are coloured differently.

Figure 1: Schematic map of the positions of CNEs around the *shox* gene. Blue CNEs are duplicated with the *shox2* gene. A blue box represent those CNEs in the Sabherwal *et al.* CNE4 region, a red box shows those CNEs in Sabherwal *et al.* CNE5 region, a cream box shows those CNEs in Sabherwal *et al.* CNE6 region, a green box shows those CNEs in Sabherwal *et al.* CNE7 region, a brown box shows those CNEs in Sabherwal *et al.* CNE8 region and a green box shows those CNEs in Sabherwal *et al.* CNE9 region.

CNEs in bold are duplicated around the s*hox2* gene.

### *shox* gene expression in zebrafish

Humans generate two isoforms of the *SHOX* gene (figure 1A), which have different expression patterns [2]. Zebrafish appears to have only one isoform (figure 1). Evidence of the zebrafish *shox* gene comes from zebrafish specific ESTs and mRNA (figure 1), as well as protein homology with mouse and human proteins. The second human isoform includes a downstream exon that does not appear, based on homology, to be present in zebrafish.

**Figure 2:**
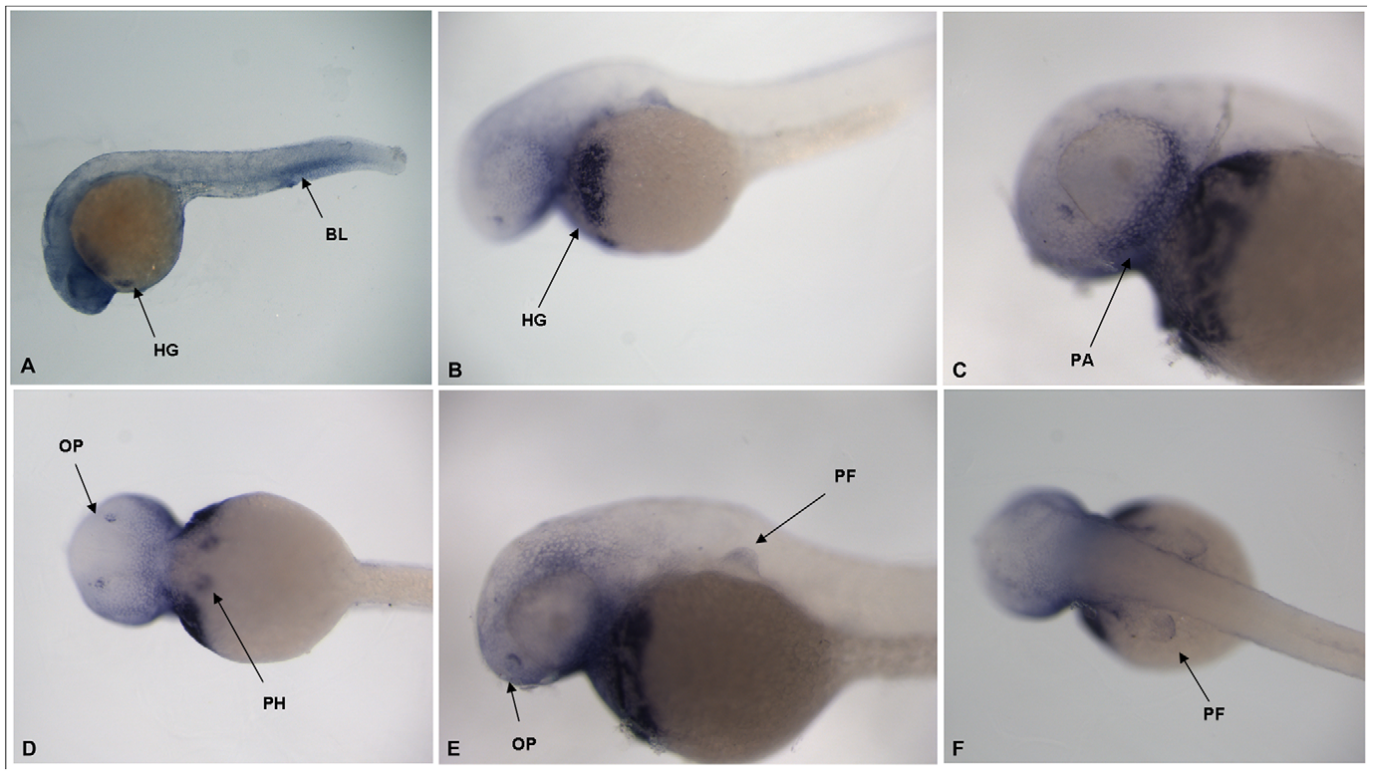
*Shox* gene expression in whole zebrafish embryos: 24 hpf embryo (HG = hatching gland) (A), view of hatching gland in 72 hpf embryo HG = hatching gland (B), close up of head region showing pharyngeal arch (PA) 72 hpf embryo (C), ventral view of 72 hpf embryo showing olfactory pits (OP) and putative heart (PH) (D), view of the pectoral fin (PF) in 72 hpf embryo (E) and dorsal view of pectoral fins (PF) in 72 hpf embryo (F).

Expression of the zebrafish *shox* gene can be detected at 24 hours post fertilisation (hpf) in the blood (BL), putative heart, hatching gland (HG) and brain, along with some non-specific expression (figure 2A). By 72 hpf, *shox* expression is more specific and can be detected in the hatching gland (HG) (figure 2B), the pharyngeal arch (PA) (figure 2C), the olfactory epithelium (OP) (figure 2D, 2E), the putative heart (PH) (figure 2D) and the fin bud apical ectodermal ridge (FB) (figure 2E, 2F).

### Comparison of enhancer activity of *Fugu* and human CNEs in zebrafish embryos

**Figure 3:**
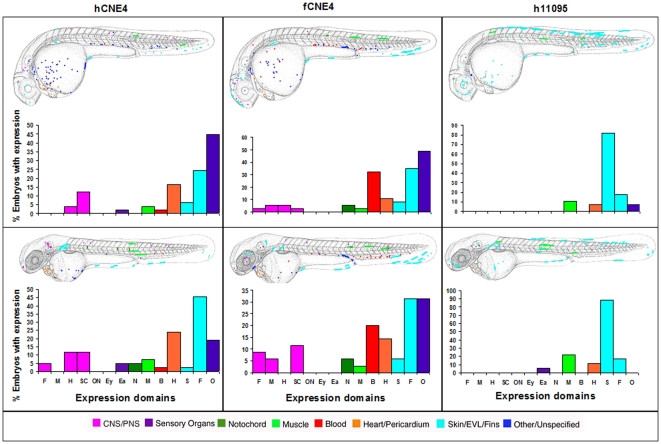
Composite overview of GFP expression patterns induced by *shox* CNE4. Expression pattern in Fugu CNE4 at day 2 (n = 37/180) and at day 3 (n = 35/174). Expression pattern in human CNE4 at day 2 and at day 3 (n = 42/236). Expression pattern in human CR00011095 day 2 (n = 28/276) and at day 3 (n = 18/249). Fugu CR00011095 showed no expression on day 2 (0/121) and day 3 (n = 0/111). F = Forebrain, M = Midbrain, H = Hindbrain, SC = Spinal cord, ON = Other Neurons, Ey = Eye, Ea = Ear, N = Notochord, M = Muscle, B = Blood, H = Heart, S = Skin, F = Fin, O = Other.

At both day 2 and day 3, Sabherwal's CNE4 drives reporter expression in fin, the cardiovascular system, brain, skin, muscle and notochord, using either human or *Fugu* elements. Additionally, the human element drives expression in the ear at day 2 and day 3 (figure 3).

**Figure 4:**
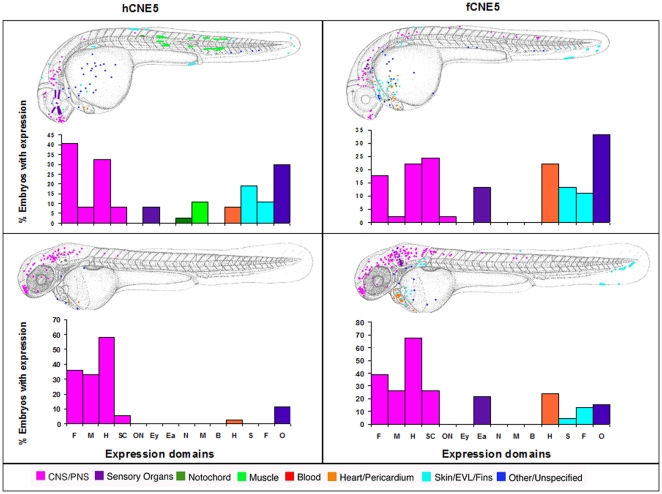
Composite overview of GFP expression patterns induced by *shox* CNE5. Expression pattern in human CNE5 at day 2 (n = 37/148) and at day 3 (n = 36/137). Expression pattern in Fugu CNE5 at day 2 (n = 45/77) and at day 3 (n = 46/64). F = Forebrain, M = Midbrain, H = Hindbrain, SC = Spinal cord, ON = Other Neurons, Ey = Eye, Ea = Ear, N = Notochord, M = Muscle, B = Blood, H = Heart, S = Skin, F = Fin, O = Other.

Both human and *Fugu* versions of Sabherwal's CNE5 drive expression in the brain, skin, heart and fins. In addition, the *Fugu* element drives some expression in the ear, while the human element drives expression in the muscle, notochord and eye (figure 4).

**Figure 5:**
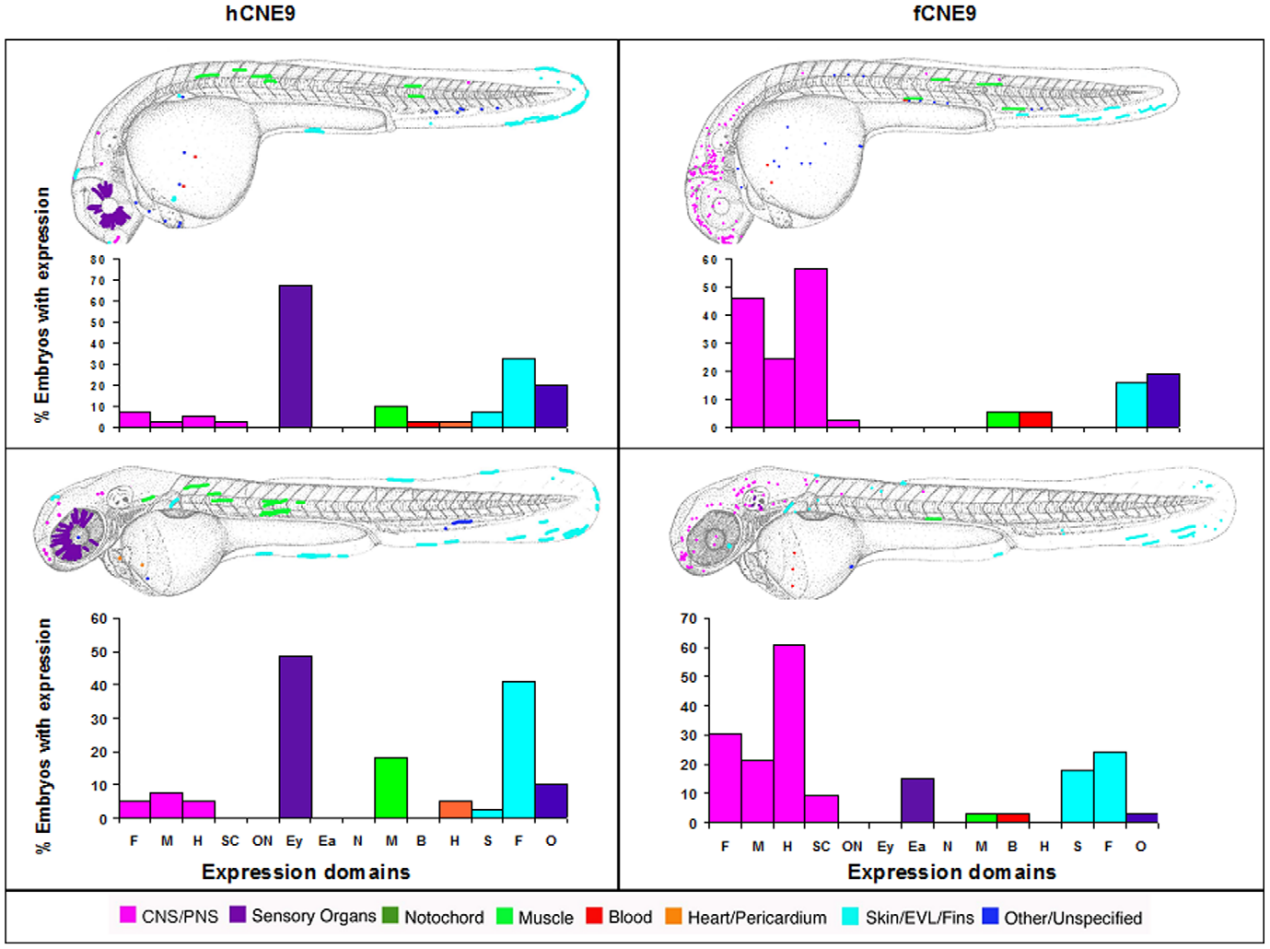
Composite overview of GFP expression patterns induced by *shox* CNE9. Expression pattern for human CNE9 at day 2 (n = 41/119) and at day 3 (n = 39/109). Expression pattern in Fugu CNE9 at day 2 (n = 37/93) and at day 3 (n = 33/91). F = Forebrain, M = Midbrain, H = Hindbrain, SC = Spinal cord, ON = Other Neurons, Ey = Eye, Ea = Ear, N = Notochord, M = Muscle, B = Blood, H = Heart, S = Skin, F = Fin, O = Other.

Sabherwal's human CNE9 predominantly drives expression in the eye at both day 2 and day 3 whereas the *Fugu* element drives expression mostly in the brain at these time points. Human and *Fugu* elements drive expression in the fin and blood (figure 5).

**Figure 6:**
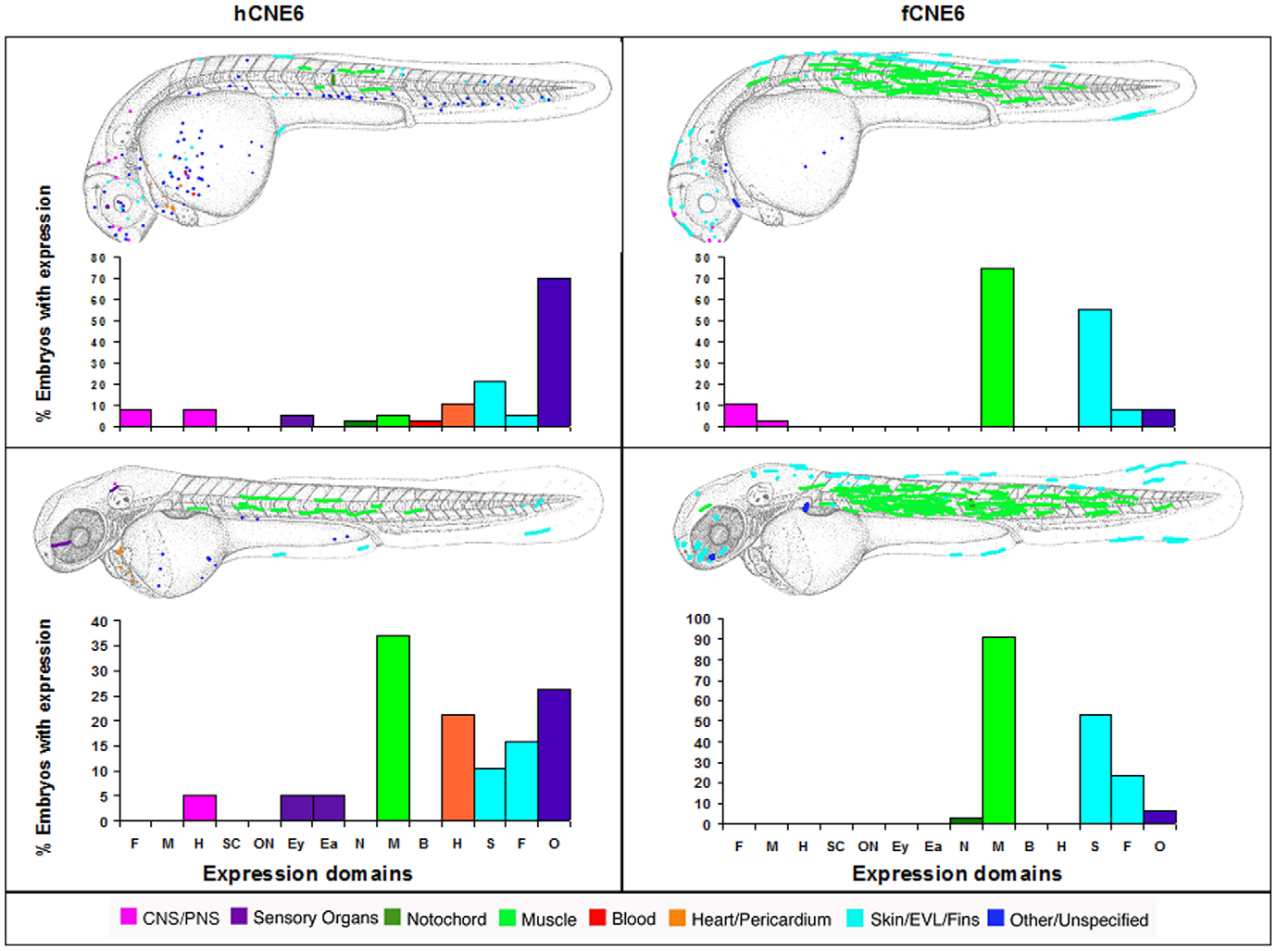
Composite overview of GFP expression patterns induced by *shox* CNE6. Expression pattern of human CNE6 at day 2 (n = 37/424) and at day 3 (n = 19/410). Expression pattern in Fugu CNE6 at day 2 (n = 36/103) and at day 3 (n = 34/78). F = Forebrain, M = Midbrain, H = Hindbrain, SC = Spinal cord, ON = Other Neurons, Ey = Eye, Ea = Ear, N = Notochord, M = Muscle, B = Blood, H = Heart, S = Skin, F = Fin, O = Other.

*Fugu* CNE6 drives expression predominantly in muscle, skin and fin, while Sabherwal's human CNE6 drives expression in a more diverse array of tissues; in particular heart and ear at day 2 and muscle, ear, eye and heart at day 3 (figure 6).

**Figure 7:**
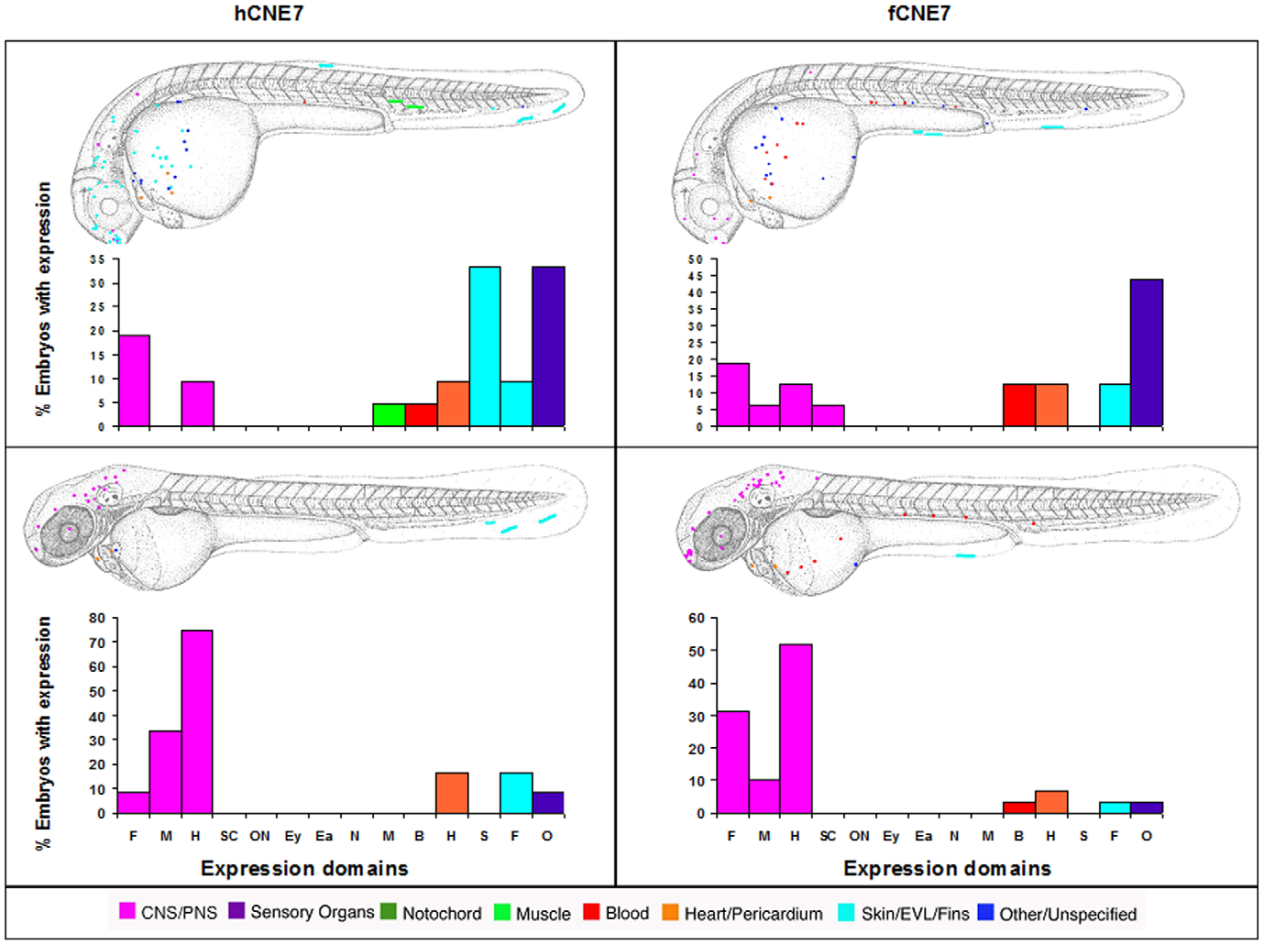
Composite overview of GFP expression patterns induced by *shox* CNE7. Expression pattern of human CNE7 at day 2 (n = 21/355) and at day 3 (n = 12/339). Expression pattern of Fugu CNE7 at day 2 (n = 16/249) and at day 3 (n = 29/227). F = Forebrain, M = Midbrain, H = Hindbrain, SC = Spinal cord, ON = Other Neurons, Ey = Eye, Ea = Ear, N = Notochord, M = Muscle, B = Blood, H = Heart, S = Skin, F = Fin, O = Other.

Sabherwal's CNE7 drives expression in brain, fin and the cardiovascular system with both human and *Fugu* versions of the CNE. Additionally human CNE7 at day 2 drives expression in the skin (figure 7).

**Figure 8:**
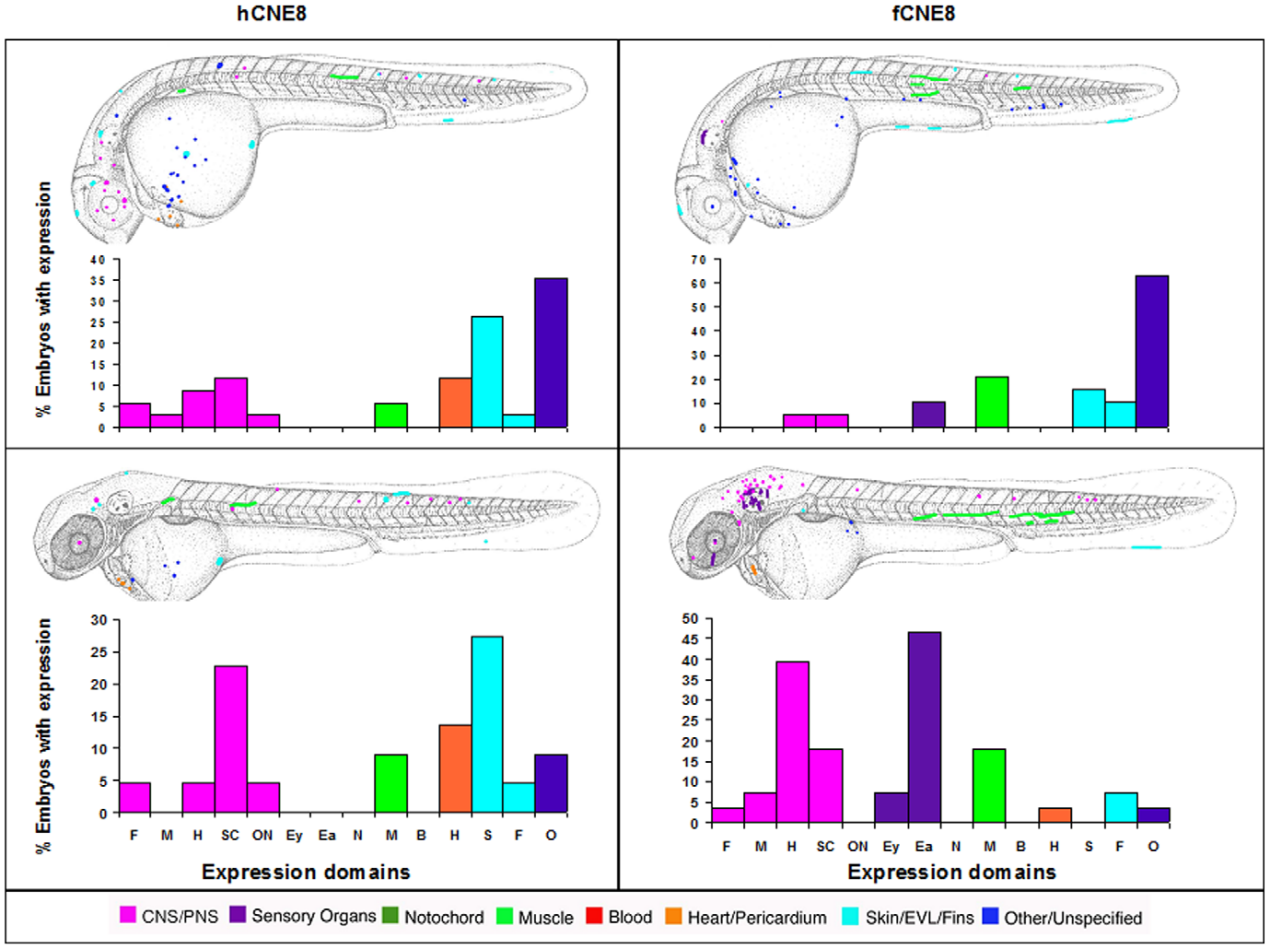
Composite overview of GFP expression patterns induced by *shox* CNE8. Expression pattern of human CNE8 at day 2 (n = 33/618) and at day 3 (n = 22/589). Expression pattern of Fugu CNE8 at day 2 (n = 19/117) and at day 3 (n = 28/107). F = Forebrain, M = Midbrain, H = Hindbrain, SC = Spinal cord, ON = Other Neurons, Ey = Eye, Ea = Ear, N = Notochord, M = Muscle, B = Blood, H = Heart, S = Skin, F = Fin, O = Other.

While Sabherwal's CNE8 drives expression in both species in skin, fin, CNS and muscle, the human element also drives expression in heart, while the *Fugu* CNE drives expression in the ear and eye (figure 8).

### Comparison of hCRCNE00011095 and fCRCNE00011095 with hCNE4 and fCNE4

From the alignments in MLAGAN, smaller, more deeply conserved elements can be identified within the larger Sabherwal regions (Table 1). We investigated whether these direct more specific expression patterns. Additionally, we hypothesised that Sabherwal's larger elements might introduce noise or CNE non-specific expression patterns.

By comparing Sabherwal's human CNE4 with the our smaller CNE hCRCNE00011095, we found that our hCRCNE000111095 drives less expression (28 out of 276 embryos at day 2, 18 out of 249 at day 3) than Sabherwal's hCNE4 (49 out of 243 embryos at day 2, 42 out of 236 embryos at day 3). Most of this expression is observed in skin, with some additional expression in fin, muscle and heart. This expression pattern is more cell type specific than Sabherwal's larger CNE (figure 3). In *Fugu*, our smaller CNE fCRCNE00011095 showed no expression at either time point (out of 121 embryos at day 2 and 111 at day 3).

### Duplicated CNEs in *shox2*

Human *SHOX* locus CNEs with CONDOR IDs CRCNE00011085, CRCNE00011098, CRCNE00011094, CRCNE00011104, CRCNE00011110 and CRCNE00011111 (table 1) are duplicated around the human *SHOX2* locus. One copy of these CNEs can also be found in rodents around the *Shox2* locus as there is no *shox* gene in these animals. This reflects the ancestral origins of not only the genes but some of their regulatory elements [13], [14]

## Discussion

Mutations in the *SHOX* gene and deletions of conserved non-coding elements (CNEs) downstream of the *SHOX* gene have been shown to be responsible for the dominantly inherited skeletal dysplasia Leri-Weill Dyschondrosteosis (LWD) [1]. Assessing the function of these CNEs relies on accurate methods of delineating the sequences involved. Goode *et al* (2011) have recently shown that short flanking sequences around a CNE can have a profound impact on CNE function and, therefore, have cautioned that CNEs should be accurately delineated before being used in studies [15]. It is therefore important to understand the limits of a CNE and how a study defines them. Sabherwal *et al.* [10] defined their CNEs by comparing the human and chicken genome and identifying regions with over 70% identity over greater than 200 bp of sequence. Six of those regions show identities of around 80% over >400 bp of sequence between human and chicken. Our analysis uses 65% identity over at least 40 bp between *Fugu* and human, resulting in the identification of shorter regions of much more deeply conserved sequence. Our results in the zebrafish embryo have shown that Sabherwal's CNE4 drives reporter expression in a variety of tissues, compared to the more deeply conserved sub-sequence, CRCNE000111095, which fails to drive expression when using the *Fugu* element, but drives primarily fin expression when using the human element. When taking into account Goode *et al.*'s caution this would suggest that the more deeply conserved sub-sequence, CRCNE000111095 may show a more accurate representation of the function of the CNE with this CNE being responsible for enhancing limb development in human but having little enhancer activity in *Fugu* [15].

When comparing these data, it should also be noted that the zebrafish whole embryo system we have employed is very different from the chick limb electroporation system used by Sabherwal *et al.* [10]. Using the whole embryo permits the identification of reporter expression in any tissue whereas electroporation of the element and promoter into the chick limb bud will only detect expression in that particular tissue or region [11]. Thus the chick system is ideal for quickly determining if an element drives expression in the limb but it does not allow for identification of where else in the animal the CNE might be functional. Rodent models have commonly been employed to identify expression in whole embryos but *SHOX* is absent from the rodent lineage. This has led us to investigate the role of *shox* and its accompanying CNEs in zebrafish.

To this end, we examined the expression pattern of the *shox* gene in 24 hpf and 72 hpf zebrafish embryos. Our results show that *shox* gene expression can be seen at 24 hpf in the putative heart, hatching gland and brain while at 72 hpf expression persists in the hatching gland (HG figure 2B) and putative heart (PH figure 2D) but is also seen in the pharyngeal arches (PA figure 2C), the olfactory epithelium (OP figure 2D, 2E) and the fin bud apical ectodermal ridge (PF figure 2F). It is interesting to note that human *SHOX* CNEs drive reporter expression in other tissues, as *shox* gene expression in the whole human embryo has only been identified in the limbs and the first pharyngeal arch [6]. However, in chick, *SHOX* gene expression is more widespread than just limb buds and pharyngeal arch and includes the neural tube, muscle and the mesenchyme overlaying the eye [7], [10].

Human, zebrafish and chick all show *SHOX* gene expression in limb, with all of the human elements, as described by Sabherwal *et al.* [10], when injected into whole zebrafish embryos, driving some fin expression. The difference between our results which show expression in not just the pectoral fin (considered to be most related to human limb [16]) but other fin structures and those of Sabherwal *et al.* can be accounted for if we separate expression seen in the pectoral fin (an example of paired fins) from that observed in the caudal fin (an example of the median fin). The development of these fins has been shown to be different, with the median fins developing directly from the epidermal fold surrounding the caudal half of the young larvae, also called the median fin fold [17]. The paired fins arise from a local proliferation of the lateral plate mesoderm to form the fin bud [16] but the exoskeleton (the fin rays) eventually develops within an epidermal fin fold, in a process that resembles the development of the median fins from the median fin fold [17]. It is therefore probably more accurate to compare the pectoral fin buds with human limb buds, as early fish fin buds and tetrapod limb buds show morphological resemblances. They also both contain similar signalling centres such as the zone of polarizing activity (ZPA) in the posterior mesenchyme, the apical ectoderm ectodermal ridge (AER) and the ventral ectoderm [16], [18]–[22]. However, the AER of the tetrapod limb progressively degenerates during development, whereas the zebrafish apical ectoderm will form an elongated fin fold in which the external part of the fin, including the fin rays will eventually develop [16], [23]. Therefore it may be more accurate when comparing our findings with those of Sabherwal *et al.* to consider all fin expression. Except that, if we do assume that the fin buds are a more appropriate model for human limb development, it can be seen that hCNE4 hCNE5 and hCNE9 are the only elements that show pectoral fin expression.

Dividing up the fin expression into paired and caudal would explain some differences between our results and those seen by Sabherwal *et al.* [10]. However, it is interesting to note that all the human CNEs we tested drive caudal fin expression. This may well be accounted for by the fact that the assay was performed in zebrafish and the regulatory machinery may be similar between paired and medial fin development in early zebrafish development. Therefore, we cannot overlook the fact that, despite their different embryonic origins, paired and median fins may utilize a common suite of developmental mechanisms. Freitas *et al.* [24] looked at lampreys, which diverged from the lineage leading to gnathostomes before the origin of paired appendages. They showed that lamprey median fins also develop from somites and express orthologous of *hox* and *tbx* genes, which are important in limb development. They suggest that the molecular mechanisms for fin development originated in the somitic mesoderm of early vertebrates, and that the origin of paired appendages was associated with redeployment of these mechanisms to lateral plate mesoderm. They argue it is possible that the mechanisms of fin and limb development were established in median fin folds, even before the origin of vertebrates [24]. In order to investigate the evolution of these CNEs, we tested homologous CNE regions from both the *Fugu* and human genomes in zebrafish embryos. From this we can see that in general, patterns of reporter gene expression in zebrafish caudal or paired fins is similar whether injecting *Fugu* or human CNEs, suggesting that CNE function in these appendages remains similar between *Fugu* and human.

In addition to the limb/fin expression seen in zebrafish, human and chick, *shox* CNEs drive expression in predominantly the same tissues that show *shox* gene expression. For example, much of the CNE expression seen in all the embryos is attributed to areas labelled as unspecified cells, which include cells of the hatching gland and the pharyngeal arch tissues, which also show *shox* gene expression in the zebrafish embryo. However, it is perhaps not surprising there should be expression in both the limb/fin and the pharyngeal arches, as these are not only the only tissues to show *shox* expression in human but Gillis *et al.* [25] have recently demonstrated that shared developmental mechanisms pattern the vertebrate gill arch and paired fin skeletons, which might explain the *shox* expression pattern seen in zebrafish and human [25].

A key area of *SHOX* gene expression, from a human disease context, is in the brain and consequently the identification of *SHOX* CNEs that drive expression in the brain (CNE4, CNE5, CNE6 and CNE7) is of significance. In clinical studies, there has been some association between Leri-Weill Dyschondrosteosis (LWD) and mental retardation. Shears *et al.* [1] noted learning disabilities in a pair of female monozygotic twins with LWD. They suggested that this might be explained by deletion of contiguous genes, since the responsible deletion encompassing the *SHOX* locus extended into the X-specific region. In Spranger *et al.* [26] a male patient with LWD, mental retardation, myoclonic epilepsy and chondrodysplasia punctata was described. Molecular mapping showed the maternally-derived deletion included *SHOX*, *ARSE* (the gene mutated in X-linked chondrodysplasia punctata) and the putative mental retardation locus *mental retardation, X-linked 49* (*MRX49*). Therefore, it is possible that the reporter expression seen in other tissues such as the brain that we see in our results (figure 4, figure 5, figure 7 and figure 8) and Sabherwal *et al.* report in their discussion might be due to these CNEs acting as regulatory inputs for other genes in the PAR1 region rather than *SHOX*. If they are indeed used only to regulate *SHOX*, it leads to the question why *SHOX* disruption in human patients does not show abnormalities in these tissues. A possible explanation is that these patients are haploinsufficient and not completely null for SHOX and that those embryos which are complete nulls are spontaneously aborted. Haploinsufficient patients might retain sufficient SHOX to form most tissues but not enough for full limb length. Robertson *et al.* [27] reported a patient homozygous for *SHOX* but who also showed mental retardation. Unfortunately one of the deletions again extended into the PAR1 region but not the X-specific region, leading to their suggestion of another possible mental retardation, X locus in the PAR1 region [27]. A later study by Zinn *et al.* [28] found that complete *SHOX* deficiency causes Langer mesomelic dysplasia; displaying a more severe form of limb shortening and dwarfism than LWD. However, the authors note that none of the Langer patients were homozygous for complete *SHOX* gene deletions. Zinn *et al.* [28] have also suggested that this discrepancy could be accounted for if none of the patients were truly null for *SHOX* activity, which could be associated with poor viability. They state that one subject is heterozygous for a complete *SHOX* deletion and a frameshift which truncates the protein after only 13 amino acids of the homeodomain and is thus likely homozygous null for SHOX [28]. Thirteen amino acids of the homeodomain would probably make the gene product long enough that there is no re-initiation further along the gene (thus no truncated but viable protein) [29] so it would make the gene subject to nonsense-mediated decay [30]. Zinn *et al.* [28] also suggested that classic Langer mesomelic dysplasia may be uncommon because most deletions extend to contiguous genes, resulting in additional phenotypes such as developmental delay. They reason that, as none of their patients had congenital anomalies apart from their skeletal features, and none had developmental delay, if *SHOX* acts outside of the musculo-skeletal system, its extra skeletal functions are redundant with other genes; perhaps *SHOX2* or other *HOX* genes.

Interestingly, our results indicate that some *shox* CNEs are duplicated at the *shox2* locus. One of the more interesting evolutionary features of *shox* is that it is missing in the rodent lineage. Looking at the anatomy of rodents, they have elongated feet rather than long bones. A possible explanation may be that *shox2* is required for limb development, while *shox* is required for the regulation of limb extension. Indeed, absence of *shox* may explain the relatively short leg length common to the rodents. In human, both *SHOX* and *SHOX2* are expressed in the limbs but as discussed in the introduction they have subtly different patterns. *shox2* in zebrafish is expressed in early diencephalon and otic vesicle, then hindbrain neurons and cranial ganglia tegmentum and tectum, ventral hindbrain neurons in subventricular zone and in marginal zone, cranial ganglia and pectoral fin (ventral part and AER) [31] while mutations in the *shox2* gene have resulted in heart abnormalities [32]. It is of note that in zebrafish, Sabherwal CNE9 drives strong brain and spinal cord expression and Sabherwal CNE5 drives heart expression. Both these CNEs have homologous sequences at the *shox2* locus, a gene that shows expression in the brain and heart, suggesting a similar role for these CNEs in regulating *shox2*, a role that may also be conserved in rodents. In addition a recent paper by Vickerman *et al* has shown that the muscles of *SHOX2* mutant mice show severe developmental abnormalities. This might explain why we see muscle expression in our analysis [33] although the CNE with the majority of muscle expression, CNE6, is not one that is duplicated at the *shox2* locus. *SHOX* has been shown to be expressed in muscle in both human and mouse [2], [7] but no muscle expression is seen in our zebrafish *in situ* data. It is unclear why this is the case but might be the due to the early developmental stages used for the *in situs*. In both mouse and human, expression of *SHOX* in the muscle was not reported until later stages of development.

More recently Durand *et al.* [34] analysed enhancer elements upstream of the *SHOX* gene using chick electroporated cornea as a comparison with the chick limb. They showed a lack of expression in the cornea, while there was expression of these elements in the limb bud. Parts of four of these regions correspond to CNEs identified through *Fugu* : mammal comparisons, two of which we screened in whole zebrafish embryos. For CRCNE00011089, corresponding to Durand's CNE2, we observed expression in the forebrain and retina. For CRCNE00011082, corresponding to Durand's CNE5, we observed reporter expression in the ear [34]. The additional expression we see in these elements compared to Durand *et al.* is likely to be due to the nature of their assay while the lack of limb expression we observe may be due to the delineation of flanking sequence between Durand's CNEs and ours [34].

The methods we have employed remove CNEs from the context of their regulatory landscape and only enhancers of function are investigated. Hence it is possible that elements, such as CNE6, which appears to drive little brain or CNS expression, may act as a repressor for some parts of the brain enhanced by the other CNEs. To understand this, a different approach and further studies must be undertaken.

### Conclusion

In conclusion we have shown that the *shox* gene is expressed in a number of tissues in the developing zebrafish embryo including fin, pharyngeal arch and brain, highlighting similarities with the expression of this gene in human and chick. We have also investigated where CNEs identified by Sabherwal *et al* drive expression in a whole embryo system, advancing our understanding of the regulatory region around this gene in an *in vivo* model and thus identifying additional tissues under the regulatory control of these CNEs. We have presented evidence that more deeply conserved sub-sequences within Sabherwal *et al.'s* CNEs, may show a more accurate representation of the function of the CNE. Lastly we have identified *shox* CNEs which are duplicated at the *shox2* locus and are conserved in the rodent lineage around the *shox2* locus. These data taken together suggest an evolutionarily important role for these CNEs in vertebrate development and will hopefully lead to a better understanding of gene regulation and the human short stature disorders such as Leri-Weill Dyschondrosteosis.

## Materials and Methods

### Identification of CNEs around the *shox* gene

Multiple alignments were constructed in MLAGAN [11] of the genomic loci surrounding the human, dog, opossum and Fugu *shox* genes. 34 CNEs were identified with at greater than 65% identity over at least 40 nucleotides. All CNEs are also identifiable in at least chimp, chick and frog. Six of the CNEs share sequence identity with CNEs at the *shox2* locus. All data is stored in a publicly accessible database, CONDOR [12], and can be retrieved from http://condor.nimr.mrc.ac.uk.

### Enhancer screen

We assayed for enhancer activity in a method adapted from Muller and colleagues [35] and described in the paper by Woolfe *et al.* [36]. The candidate enhancer elements were the six human conserved non-coding elements that had been identified by Sabherwal *et al.* [10] around the *SHOX* gene, three of which (CNE4, 5 and 9) had shown enhancer activity in the chick limb. We injected these regions, and the same regions from *Fugu*, into zebrafish embryos to look at their expression in a whole embryo system. We also looked at a smaller, more conserved element (CRCNE00011095) within the CNE4 region, using both human and *Fugu* DNA.

### PCR

CNEs and negative controls were PCR-amplified from *Fugu* and human genomic DNA. Primers were as follows: [human CNE4 Forward TTTTGCAGTGTTATGCACTCG, human CNE4 Reverse CCCCCTCTTAGTCCTGGTGT]; [human CNE5 Forward GCCTCCCTCGGGAGCGATTGTATCTATT, human CNE5 Reverse CATCCTCATCCTGCCTTCGAAGGCAGAC]; [human CNE6 Forward TCGTCGTCATACTGTCACTGG, human CNE6 Reverse TACCCTAAGCCCTTCCTTCC]; [human CNE7 Forward GAGGCTGCAGCTCACCCCGC, human CNE7 Reverse AAACTGCACAGACCAGGTCT]; [human CNE8 Forward TCCCCTCTGAGCCTGGCAGG, human CNE8 Reverse CTCCATATCCCTGCAGAGAC]; [human CNE9 Forward TCCCCCTATACTTTACTTCTTTGC and human CNE9 Reverse GCCTCTTGTGTCTGCAGTGT]; [*Fugu* CNE4 Forward TGGTTAACGATAGATTCTTG, *Fugu* CNE4 Reverse GTCATGTGTCATTCATTCAC]; [human CNE5 Forward GCCTCCCTCGGGAGCGATTGTATCTATT, human CNE5 Reverse CATCCTCATCCTGCCTTCGAAGGCAGAC]; [*Fugu* CNE5 Forward ATTTTTCATCGCCCTTGTTG, *Fugu* CNE5 Reverse AACAAAGAGCGGGAGAGTGA]; [*Fugu* CNE6 Forward CCTAAATTACAGTTTTCTCTTTGACTC, *Fugu* CNE6 Reverse TTACAGTTTTCTCTTTGACTC]; [*Fugu* CNE7 Forward ACCTCCCGACCTCCAAACT, *Fugu* CNE7 Reverse CCAACACTTTCTCTGTCTTTGC]; [*Fugu* CNE8 Forward CCACCATGTATATCTTATAATG, *Fugu* CNE8 Reverse GCACCTCCTATATATTTAAA]; [*Fugu* CNE9 Forward GTTCCATTCTCTGTCAAGGTCTG, *Fugu* CNE9 Reverse ACGCGTATGTAAATGGATCCTTT]. For the more conserved CRCNE00011095 region primers were: [human CRCNE00011095 Forward ATTTGCCTTTTAATGGGGTGT, human CRCNE00011095 Reverse GTCTTCATTGATTCCGCAGAAAG]; [*Fugu* CRCNE00011095 Forward CACACCTTCTCAGCCTTCCT, *Fugu* CRCNE00011095 Reverse CACGGCGATTAAGTTTGTGG].

### Microinjection

Element DNA or control DNA (at 150–300 ng/µl), reporter construct DNA consisting of EGFP (Clontech, Palo Alto, California, United States) under the control of a minimal promoter from the mouse β-globin gene (at 25 ng/µl), and phenol red (at 0.1%, used as a tracer) were combined and co-injected into embryos between the one-cell stage and four-cell stage.

### Screening

Any embryos developing abnormally were discarded before screening. The embryos were treated with 1-phenyl-2-thiourea (PTU) and screened for GFP activity on the second (approximately 26–33 hpf) and third (approximately 48–55 hpf) days of development. GFP-expressing cells were classified according to the following tissue categories: forebrain, midbrain, hindbrain, spinal cord, eye, ear, notochord, muscle, blood, heart or fins. Cells that did not fall into one of these categories (or that were not possible to identify from morphology or localisation) were categorised as “other" or “unspecified". The location and tissue category of each GFP-expressing cell for each embryo was recorded onto an overlay of a camera lucida drawing of a 31 hpf and a 50 hpf embryo. GFP expression data was collected from between 20 and 50 expressing embryos per element injected. Cumulative, overlaid, schematised expression data for each element was compiled into a single JPEG file, giving an overall representation of the spatial pattern of each element. The number of cells per tissue in which GFP expression was detected was graphed giving an indication of the strength of the element's enhancing properties.

### *In Situ* hybridisation of *shox* gene

*In Situ* hybridisation of *shox* gene was carried out using the Thisse's method as published on Zfin [37]. Probes were made to the first and last exons of the *shox* gene using the Thisse's PCR method B [37] with the following primers: [*shox* 1st exon Forward AAACCCTTCTCCACGCAAA, *shox* 1st exon T7 Reverse TAATACGACTCACTATAGGGAGAAGCCCTTGTCACGCTAA]; [*shox* last exon Forward AACACACTCCCATCCTCACC, *shox* last exon T7 Reverse TAATACGACTCACTATAGGGTTGTTTTGTTTTAACTGTGAGTGTCA]. Embryos were treated with 1-phenyl-2-thiourea (PTU), manually dechorionated and fixed in 4% paraformaldehyde at 24 hpf and 72 hpf.

### Zebrafish care

Zebrafish embryos were obtained from sibling crosses from adult fish housed at the fish facility at Queen Mary University of London. Zebrafish were raised and bred and embryos staged following standard protocols [38], [39]; stages are described as the approximate number of hours post-fertilisation (hpf) when embryos are raised at 28.5°C. To prevent pigment formation, embryos were raised in 0.003% phenylthiocarbamide in embryo medium from tailbud stage.
